# Society Meetings

**Published:** 1904-03

**Authors:** 


### SOCIETY MEETINGS.

The American Medico-Psychological Association will hold its next annual meeting at Saint Louis, May 30, to June 3, 1904, under the presidency of Dr. A. E. Macdonald, of New York. Among the papers to be presented are the following: The alcoholic psychoses, by Henry P. Frost, Buffalo; The German psychiatric clinics, by Edward N. Brush, Baltimore; A case of sleep talking, by D. R. Burrell, Canandaigua, N. Y. The headquarters of the association will be at the Planters Hotel. For further particulars address the secretary, C. B. Burr, Flint, Mich.

The first French Congress of Climotothetherapy and Hygiene of Towns will be held at Nice, April 4-9, 1904, under the presidency of Professor Chantemesse, as heretofore announced. Reductions in railway fare in France at the rate of 50 per cent. will be allowed. A number of excursions and receptions have been provided. For further information, apply to Dr. Hérard de Bessé, general secretary, Beaulieu Sur. Mer.

---

The Medical Society of the Missouri Valley will hold its next meeting at Lincoln, Neb., March 24 and 25, 1904, under the presidency of Dr. A. D. Wilkinson, of Omaha. Dr. Charles Wood Fassett, Saint Joseph, is the secretary.

---

The tenth quadrennial Congress of Polish Physicians and Scientists will be held at Lemberg, Austria, July 20-24, 1904. Prof. E. Machok is the chairman of the committee of arrangements, and Prof. W. Sieradzki, secretary. Dr. Francis E. Fronczak, of Buffalo, is the representative of the committee for the United States.

---

The Vanderburgh County (Ind.) Medical Society held its regular meeting January 19, 1904, at the offices of Drs. Walker and Welborn, it being the tenth anniversary of the opening of the Evansville Sanitarium. The meeting took the form of a reception, at which luncheon was served and was much enjoyed.

This society three years ago took into its membership all the homeopaths and eclectics, probably the first in the country to take such sweeping an action. The results, however, have fully justified its course. It is remarkable that a city of 75,000 inhabitants exists without a doctor's sign displaying the words "homeopath" or "eclectic." Practically every physician in the county is a member of the regular organisation.

---

The National Association of United States Pension Examining Surgeons will hold its third annual meeting at Atlantic City, N. J., June 6 and 7, 1904, under the presidency of Dr. William A. Howe, of Phelps, N. Y. The program committee, consisting of William A. Howe, president; Charles H. Glidden, treasurer, and Wheelock Rider, secretary, makes the following suggestions for titles of papers and subjects for discussion:

Pain—Its physical diagnosis.

The malingerer—A symposium. (The program committee will arrange this symposium if members who are doing special work will volunteer, each in his own line.)

Disease as a disabling factor in earning capacity.

Novel methods of physical diagnosis.

Old age as a modifying factor in disease and disability.

Nervous diseases as disabilities.

Tricks of the pension examiner's trade—Wednesday experiences.

These titles are intended to be merely illustrative of the general character of the papers which the committee thinks most likely to be interesting and useful. Intending readers may select other titles or may modify these in any way which seems best, or may use them as they stand.

Papers, however, only upon subjects of special interest to pension examining surgeons are desired. A provisional program will be issued at a later date.

---

THE Buffalo Academy of Medicine held meetings during the months of January and February as follows:

*Section on Obstetrics.*—Tuesday evening, January 26. Program: Rupture of the uterus, Ludwig Schroeter; discussion opened by Matthew D. Mann.

*Section on Surgery.*—Tuesday evening, February 2. Program: Diagnosis and treatment of diseases of urinary bladder, James Henry Dowd.

*Section on Medicine.*—Tuesday evening, February 9. Program: Tetany and its relation to gastric diseases, Allen A. Jones; discussion opened by James W. Putnam.

*Section on Pathology.*—Tuesday evening, February 16. Program: Transplantation of suprarenal glands, F. C. Busch; Changes in the blood at high altitudes, C. Van Bergen. Specimens were exhibited by Drs. A. E. Woehnert, N. G. Russell, Irving P. Lyon, and W. H. Mosher.

*Section on Obstetrics.*—Tuesday evening, February 23. Program: The technic of a radical abdominal operation for carcinoma of the uterus, Wm. R. Pryor, Professor of Gynecology, New York City Post Graduate School and Polyclinic.

